# *In vitro* evaluation of the anti-diabetic potential of *Helichrysum petiolare* Hilliard & B.L. Burtt using HepG2 (C3A) and L6 cell lines

**DOI:** 10.12688/f1000research.26855.2

**Published:** 2021-04-01

**Authors:** Adebowale Emmanuel Aladejana, Graeme Bradley, Anthony Jide Afolayan

**Affiliations:** 1 Department of Biochemistry and Microbiology, University of Fort Hare, Alice, Eastern Cape, 5700, South Africa; 2 Medicinal Plant and Economic Development (MPED) Research Centre, University of Fort Hare, Alice, Eastern Cape, 5700, South Africa

**Keywords:** Helichrysum petiolare Hilliard & B.L. Burtt, myocytes, α–amylase, α-glucosidase, hepatocytes

## Abstract

**Background:** *Helichrysum petiolare* Hilliard & B.L. Burtt has been listed in a survey of plants used in traditional medicine for the treatment of type 2 diabetes in the Eastern Cape of South Africa. In this study, the antidiabetic potentials of ethanol, cold aqueous (CAQ) and boiled aqueous (BAQ) extracts of *H. petiolare* were investigated.

**Methods: **The cytotoxic and glucose utilization effects of the extracts were evaluated using L6 myocytes and HepG2 (C3A) hepatocytes. α-amylase, α-glucosidase and lipase inhibition assays were also carried out.

**Results: **The ethanol extract showed significant cytotoxic effects in the treated cells. Both BAQ and CAQ extracts significantly increased glucose uptake in L6 and C3A cell lines. The CAQ extract enhanced glucose uptake more in the L6 myocytes than in the C3A cell-lines hepatocytes. The BAQ extract showed higher levels of inhibition on α–amylase and α-glucosidase than CAQ. The activities were not significantly different from acarbose. However, BAQ showed lower lipase inhibition than acarbose (p<0.05).

**Conclusions: **The BAQ and CAQ extracts of *H. petiolare* may, therefore, contain pharmacologically active and relatively non-toxic hypoglycaemic chemicals, which may be effective substitutes in the treatment of diabetes mellitus.

## Introduction

Diabetes mellitus (DM) is a complex multi-factorial metabolic anomaly characterized by glucose intolerance and hyperglycaemia, either due to relatively impaired insulin secretion or reduced effectiveness of insulin in facilitating glucose uptake ^1^. DM can be categorized based on its aetiology and clinical symptoms into type 1 (insulin-dependent) diabetes mellitus (IDDM), and type 2 (non-insulin-dependent) diabetes mellitus (NIDDM). Type 2 diabetes mellitus is the foremost predominant form of diabetes and it is characterized by hyperglycaemia and abnormal blood lipid levels. The leading cause of this disease has been reported to be either congenital or unhealthy lifestyle or both ^2^.

The worldwide prevalence of diabetes has increased drastically in the last 30 years, and the current statistics suggest the number is to double in the next 20 years ^3^. According to the International Diabetes Federation (IDF), the disease influences well over 366 million (M) individuals around the world which, by 2030, is projected to increase to 552 M ^4^. Nigeria with 3.2 M cases, South Africa with 2 M, Kenya with over 0.7 M, and Cameroon with over 0.5 M have been estimated to have the most elevated predominance of the disease in each sub-region of Africa ^4^.

The liver and the skeletal muscle are some of the major tissues responsible for the maintenance of the body’s glucose homeostasis. Glucose gains access into the hepatocytes and the myocytes through GLUT2 and GLUT4 receptors, respectively. The GLUT2 receptor is a transmembrane carrier protein that enables protein facilitated glucose transport into the hepatocytes; it does not need insulin. The GLUT4 receptor, however, needs to be translocated through insulin activation from the endoplasmic reticulum to the plasma membrane to facilitate glucose uptake. A postprandial spike in blood glucose is accompanied by increased insulin secretion by the pancreas. The liver, under the influence of insulin, takes up postprandial glucose and stores it as glycogen through glycogenesis. High level of blood insulin, therefore, favours increased liver uptake of blood glucose for glycogenesis. A defect in hepatic glucose uptake and GLUT4 activity may result in insulin resistance, ultimately leading to the development of diabetes. Therefore, enhanced muscle and liver glucose uptake are considered primary therapeutic targets for diabetes management ^5^.

Prevention of diabetes through healthy lifestyles (e.g. healthy diet, increased activity, weight reduction, etc.) is better than a cure. To date, diabetes has no cure, and the common oral hypoglycaemic synthetic drugs currently used in its management are accompanied by inalienable side effects ^6^. Researchers are now in search of alternative and complementary drugs to proffer enduring solutions to this metabolic disease, with limited side effects ^4^. Many herbal remedies are effective anti-hyperglycemic agents and they are also a very important component of the health care conveyance framework in most African nations ^7^. In one of their submissions, the World Health Organization (WHO) supported the assessment of medicinal plants (MPs) based on their viability, low cost and possession of very few adverse effects ^8^. Plants are used for therapeutic purposes by almost 80% of South Africans; this is because of the non-affordability of orthodox medicine and rising costs conventional treatments ^4^. However, not enough studies have been done to comprehensively evaluate the dosage, safety and anti-hyperglycemic potentials of most of the medicinal plants. There is, therefore, need to scientifically evaluate their toxicities and antidiabetic potentials through *in vivo* and/or *in vitro* studies before exposure to human subjects.

So far, surveys have reported over 44 plants in the Eastern Cape of South Africa used in traditional medicine to treat type 2 diabetes ^9, 10^. One of these herbal plants is *Helichrysum petiolare* Hilliard & B.L. Burtt, ** a plant commonly found in the Eastern Cape of South Africa, and traditionally known as *imphepho* ^9, 10^. A previous study has revealed *H. petiolare* as useful in herbal medicine for the treatment of diabetes, headache, reproductive problems, heart problems, respiratory infections, fever, high blood pressure, pain, and wounds ^11^. The whole plant is pulverised, boiled and taken as an infusion ^9^. According to the study, extracts and compounds obtained from *H. petiolare* possess antigenotoxic, antityrosinase, antioxidant, anti-inflammatory, and cytotoxic activities ^11^. To date, very little is known of the safety and mechanism of action of *H. petiolare*. This study, therefore, evaluated the toxicity and anti-diabetic potentials of several whole plant extracts of *H. petiolare* using *in vitro* methods, intending to shed light on its mechanism of action.

## Methods

### Sample collection

The whole plant of *Helichrysum petiolare* was procured from a Rastafarian who collected it from Hogsback, in Raymond Mhlaba Municipality of Eastern Cape. The purchased plant was identified and authenticated by Professor C.N. Cupido of the Department of Botany, University of Fort Hare, Alice, and indexed at the Giffen Herbarium (Voucher no. HEL-2397), University of Fort Hare, Alice Campus, Eastern Cape, South Africa.

### Preparation of ethanol, boiled and cold aqueous extracts

The whole plant of *H. petiolare* was washed, cleaned and oven-dried at 40°C. The dried sample was pulverized using an electrical blender and sieved (20 μm mesh). A portion (200 g) of the sample was then soaked individually in 1 L of 99.9% ethanol and water (for cold aqueous (CAQ) extracts) and shaken on an orbital shaker for 24 hours, while another portion was boiled in 1 L of water (for boiled aqueous (BAQ) extract) for 15 minutes. The solution was then filtered using the Buchner funnel and Whatman No. 1 filter paper and concentrated at 78°C (for ethanol (ETQ) extract) using a Rotary vacuum evaporator (Scietek, Model: RE 300), while the concentration of the aqueous extracts was done using a freeze drier (Vir Tis benchtop K, Vir Tis Co., Gardiner, NY, USA). The process yielded 18.57 g of ETQ, 15.52 g of CAQ, and 17.63 g of BAQ extracts. The concentrated extracts were stored at 4°C in the refrigerator until required ^12^.

### Reagents

HepG2 (C3A) hepatocytes (catalogue number: HB-8065) and L6 myocytes (catalogue number: JCRB9081) were purchased from Cellonex, South Africa. Fetal calf serum (FCS), Roswell Park Memorial Institute (RPMI) 1640 Medium, 3-(4,5-Dimethylthiazol-2-yl)-2,5-diphenyltetrazolium bromide (MTT), and Dulbecco's Modified Eagle Medium (DMEM) were procured from GE Healthcare Life Sciences (Logan, UT, USA). Dimethyl sulfoxide (DMSO) and phosphate-buffered saline (PBS) (with and without Ca2+ and Mg2+ and trypsin) were purchased from Lonza (Walkersville, MD, USA). Bovine serum albumin (BSA), and insulin were purchased from Sigma (St. Louis, MO, USA).

### Cell culture maintenance

Incubation of all cell cultures in a humidified environment with 5% CO _2_ was performed at 37°C. Growth medium that consisted of RPMI 1640 medium and 10% fetal calf serum was added to the HepG2 cells every 2–3 days, and the L6 myoblast cells were cultivated in an antibiotic-free media containing RPMI 1640 and 10% fetal calf serum. The cell lines were subcultured post 90% confluence.

### MTT cytotoxicity assay

To determine cell viability, 100 µl MTT (0.5 mg/ml in DMEM) was added to each well and incubated at 37°C for an hour. The MTT solution was removed, and 200 µl DMSO was added. The mixture was again incubated for 20 minutes at room temperature, and the absorbance was read at 540 nm. The cytotoxicity was expressed as a percentage of untreated control ^13^.

### Glucose utilisation assay

Using the method described by Van de Venter *et al.* (2008), L6 myocytes and HepG2 (C3A) hepatocytes were each seeded at a density of 5000 cells/well into separate 96-well plates ^14^; a row was kept devoid of cells in each plate as the “no cell control.” The L6 cell medium was later replaced with differentiation medium (DMEM containing 2% horse serum) after approximately 80% confluence and then incubated for an additional five days to allow full differentiation. The C3A cells were also incubated for an additional 2-3 days to become confluent. The spent medium of both plates was removed and substituted with fresh medium containing the extract at 200 µl/well. Treatments were continued for 48 hours, after which the spent culture medium was aspirated and the cells were rinsed once with PBS. 50 µl of 8 mM glucose solution (RPMI medium diluted with PBS and supplemented with BSA to a final concentration of 0.1%) were added, while 1000 ng insulin and 1000 ng of metformin were also added for the L6 and C3A positive controls, respectively. The plates were returned to the incubator for 2 hours (L6 cells) or 3 hours (C3A cells), after which 5 µl was transferred from them to new plates and 200 µl glucose assay reagent (glucose oxidase/peroxidase colourimetric reagent) was added. The mixtures were again incubated at 37°C for 10–20 minutes and the absorbances were measured at 510 nm using a BioTek SynergyMx UV Spectrophotometer. Estimations of glucose utilisation were done as the differences between the test samples and the no cell controls and expressed as percentages of the untreated controls. Cell viability was determined in each well using the MTT assay ^13^.

### α-amylase inhibition assay

The α-amylase assay was carried out using the 3,5-dinitrosalicylic acid (DNSA) method described by Wickramaratne *et al.* (2016) ^15^. The plant extract was dissolved in at least 10% DMSO and dissolution was further achieved in a buffer (pH 6.9, Na _2_HPO _4_/NaH _2_PO _4_ [0.02 M], NaCl [0.006 M]) at 10 to 1000 μg/ml concentrations. A 200 μl volume of α-amylase (2 units/ml) was mixed with 200 μl extract and incubated at 30°C for 10 minutes. Thereafter, 200 μl of the starch solution (1% in water (w/v)) was added to each tube and incubated for 3 minutes. 200 μl DNSA reagent (12 g of sodium potassium tartrate tetrahydrate in 8.0 mL of 2 M NaOH and 20 mL of 96 mM of 3,5-dinitrosalicylic acid solution) was applied to terminate the reaction and the mixture was steamed in an 85–90°C water bath for 10 minutes. The blend was cooled down to optimum temperature in a 37 ºC water bath and mixed with 5 ml of distilled water, and a UV Spectrophotometer (BioTek SynergyMx) was used to measure the absorbance at 540 nm. The blank with 100% enzyme activity was prepared with 200 μL of the buffer in place of the plant extract. Similarly, prepared was a sample blank, using the plant extract at each concentration in the absence of the enzyme solution. A positive control sample was prepared using acarbose (100 μg/ml–2 μg/ml) and the reaction was performed similarly to the reaction with plant extract as mentioned above. The inhibitory activity of α-amylase was expressed as a % inhibition and measured based on the below equation. The % inhibition of α-amylase was plotted against the concentrations of the extract and IC _50_ values were taken from the graph.

$$\%\;\text{α}\;\text{amylase}\;inhibition=\left(100\;x\frac{Abs_{100{\%}_{control}\;}-Abs_{Sample}}{Abs_{100{\%}_{control}\;}}\right)\;(1)$$

### α-glucosidase inhibition assay

The procedure described by Sagbo *et al.* (2018) was used for the α-glucosidase inhibition assay. Summarily, 5 μl of the plant extract (prepared at a concentration of 50 µg/ml, 100 µg/ml, and 200 µg/ml) was applied to 20 μl solution of 50 μg / ml α-glucosidase in a 96-well plate. A 60 μl potassium phosphate buffer (pH 6.8) of 67 mM was added and the plate was further incubated for 5 minutes. 10 μl of 10 mM ρ-nitro-phenyl-α-D-glucoside (PNPGLUC), was added after incubation and incubated again at 37°C for 20 minutes. 25 µl of 100 mM Na _2_CO _3_ (sodium carbonate) solution was applied after incubation and the absorption measured at 405 nm using a UV-visible spectrophotometer (BioTek SynergyMx). In addition, an enzyme blank and sample blank are prepared by adding 5 μl of deionized water instead of plant extract and 20 μl of deionized water instead of the enzyme. The spectrophotometer was zeroed using the sample blank. Acarbose was used as a positive control and the percentage inhibition was determined by the following equation:

$$\%\;\text{inhibition}=\left(1\;-\;\frac{\text{The}\;\text{absorbance}\;\text{of}\;\text{the}\;\text{test}\;\text{well}}{\text{The}\;\text{absorbance}\;\text{of}\;\text{the}\;\text{enzyme}\;\text{blank}}\;\times \;100\right)\;(2)$$

### Pancreatic lipase inhibition

Using the method described by Jaradat *et al.* (2017), 0.8 ml of 1 mg/ml porcine pancreatic lipase (prepared immediately before use in 50mM Tris-HCl buffer (pH 7.4)) was added to test tubes containing 0.2 ml of the various concentrations of plant extract (1 mg/ml stock solution in 10% DMSO, diluted to 30, 60, 120, 240, and 480 µg/ml) ^16^. The resulting mixtures were incubated for 15 minutes at 25°C. Exactly 0.1 ml of PNPB solution (20.9 mg of PNPB in 2 ml of acetonitrile) was added to each test tube after incubation, and the mixture was incubated again at 37°C for 30 minutes. Determination of pancreatic lipase activity was made by measuring the hydrolysis of p-nitro phenylbutyrate to p-nitrophenol at 405 nm using a UV-visible spectrophotometer (BioTek SynergyMx). The same procedure was repeated for the aqueous and organic extracts and for acarbose (a positive control) using the same concentrations as mentioned above. The tests were done in triplicates.

### Statistics

The obtained data were analysed for statistical significance using minitab-17 software. All experiments were repeated at least three times using different transfer numbers of C3A and L6 cells. The two-tailed Student t-test for two samples assuming equal variance was used to determine the statistical significance of each treatment. Error bars indicate SD of individual experiments, performed in quadruplicate.

## Results

### Glucose utilisation assay using L6 myocytes

**Figure 1.**
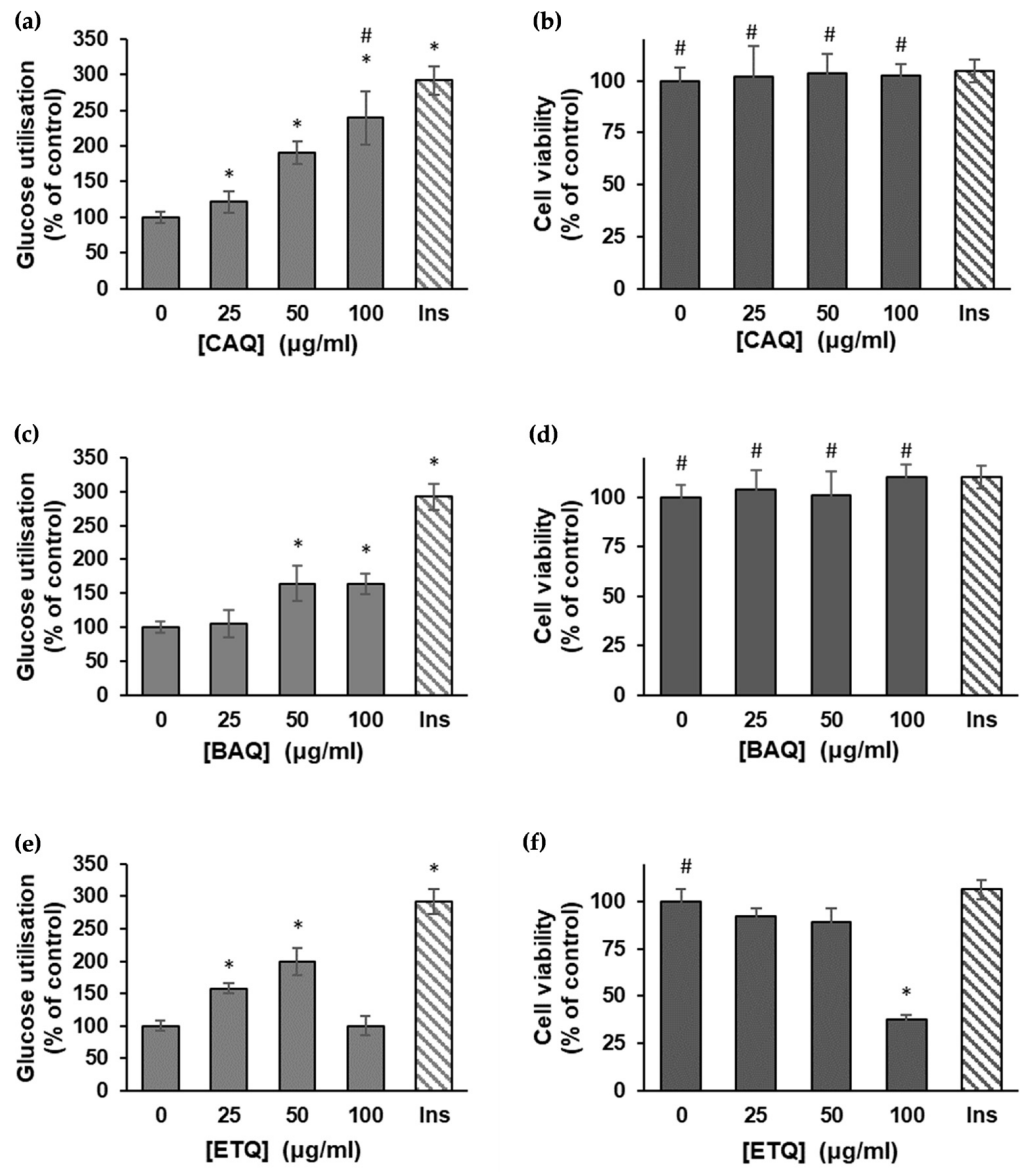
( **a**) Effect of the CAQ extract of *H. petiolare* on glucose uptake in L6 myocytes (data are expressed as mean ± SD, n = 3); ( **b**) MTT cytotoxicity of the plant’s CAQ extract on L6 myocytes (data are expressed as % of control ± SD, n = 3); ( **c**) effect of the BAQ extract of *H. petiolare* on glucose uptake in L6 myocytes (data are expressed as mean ± SD, n = 3); ( **d**) MTT cytotoxicity of the plant’s BAQ extract on L6 myocytes (data are expressed as % of control ± SD, n = 3); ( **e**) effect of the ethanol extract of *H. petiolare* on glucose uptake in L6 myocytes (data are expressed as mean ± SD, n = 3); ( **f**) MTT cytotoxicity of the plant’s ethanol extract on L6 myocytes (data are expressed as % of control ± SD, n = 3). * Indicates a significant difference (p < 0.05) compared to the untreated control, and # indicates no significant difference relative to the positive control (insulin). BAQ, boiled aqueous; CAQ, cold aqueous; ETQ, ethanol; Ins, insulin.

The results of glucose utilization assays using the L6 muscle cells are shown in Figure 1. According to the results ^17^, the CAQ extract, compared to the untreated and insulin-treated controls, induced a significant (p < 0.05) dose-dependent increase in glucose uptake across all the concentrations ( Figure 1a). The effect of the BAQ extract on glucose uptake was not dose-dependent; it failed to induce any significant change at concentrations beyond 50 μg/ml ( Figure 1c). The ethanol extract had a similar effect on the L6 cells’ glucose uptake as the CAQ, except at 100 μg/ml, where the rate of uptake plummets by roughly 50% ( Figure 1e).

The MTT assay showed the CAQ and BAQ extracts, when compared to the untreated and insulin-treated controls, had no significant toxic effect on the viability of the L6 cells ( Figures 1b and 1d). The ethanol extract, however, showed significant toxicity at 100 μg/ml, reducing the viability of the L6 cells by more than half.

### Glucose utilisation in HepG2 (C3A) hepatocytes

**Figure 2.**
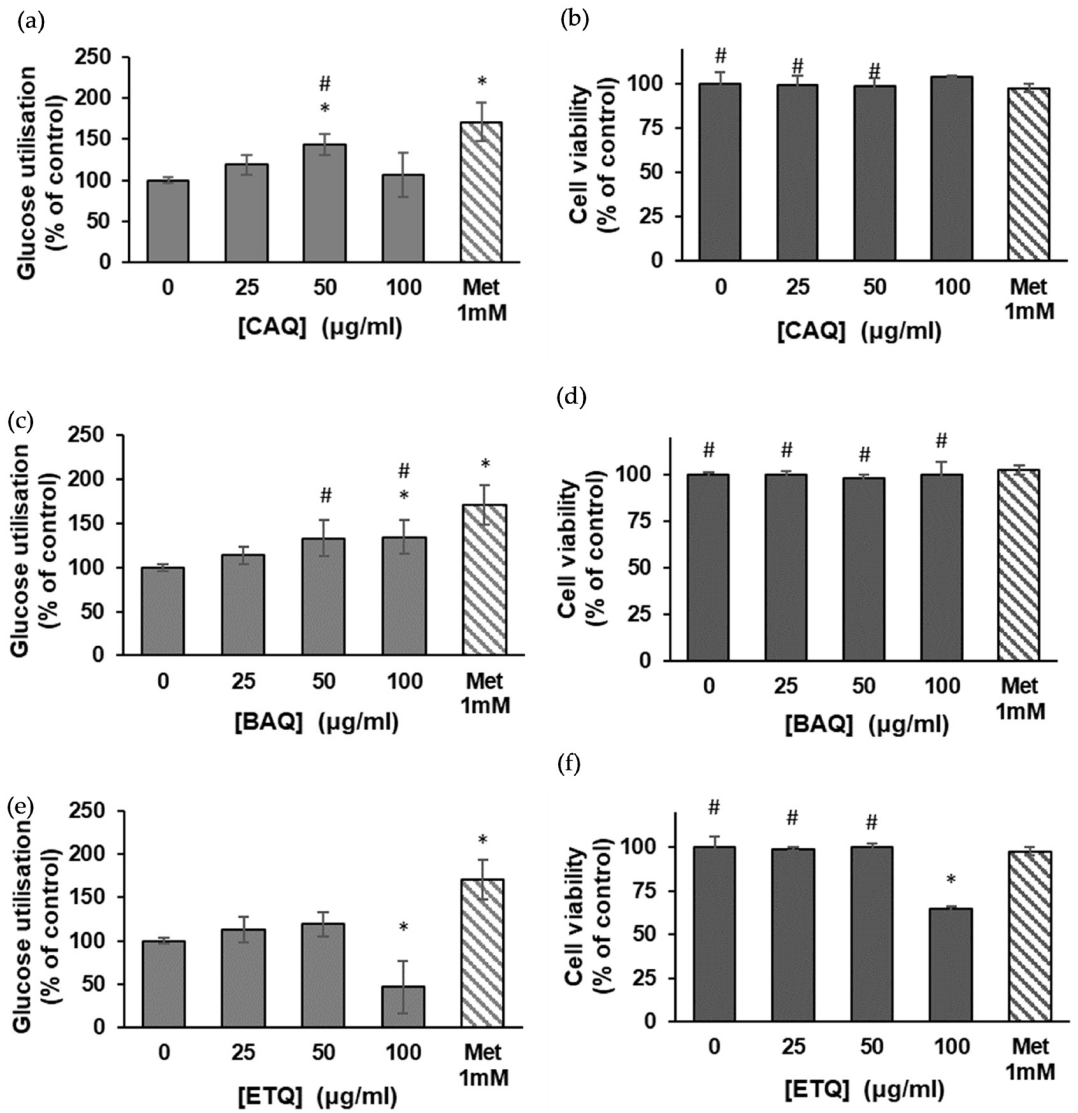
( **a**) Effect of the CAQ extract of *H. petiolare* on glucose uptake in HepG2 (C3A) hepatocytes (data are expressed as mean ± SD, n = 3); ( **b**) MTT cytotoxicity of the plant’s CAQ extract on HepG2 cells (data are expressed as % of control ± SD, n = 3); ( **c**) effect of the BAQ extract of *H. petiolare* on glucose uptake in HepG2 (C3A) hepatocytes (data are expressed as mean ± SD, n = 3); ( **d**) MTT cytotoxicity of the plant’s BAQ extract on HepG2 cells (data are expressed as % of control ± SD, n = 3); ( **e**) effect of the ethanol extract of *H. petiolare* on glucose uptake in HepG2 (C3A) hepatocytes (data are expressed as mean ± SD, n = 3); ( **f**) MTT cytotoxicity of the plant’s ethanol extract on HepG2 cells (data are expressed as % of control ± SD, n = 3). * Indicates a significant difference (p < 0.05) compared to the untreated control, and # indicates no significant difference relative to the positive control (metformin). BAQ, boiled aqueous; CAQ, cold aqueous; ETQ, ethanol; Met, metformin.

Figure 2 shows the results of glucose utilization assays using HepG2 (C3A) hepatocytes ^17^. The CAQ treated cells showed significant dose-dependent increases in glucose uptake up to 50 μg/ml; the rate, however, dropped steeply by 26% at 100 μg/ml ( Figure 2a). The result also showed concentration-dependent increases in glucose uptake in the BAQ treated cells ( Figure 2c). These increases were significant (p < 0.05) at 50 μg/ml when compared to the metformin-treated control and also significant at 100 μg/ml when compared to the untreated and metformin-treated controls; the rate of glucose utilization appeared to slow down with the increase in concentration. No significant increase in glucose uptake was observed in the ethanol-treated cells across all concentrations ( Figure 2e); in fact, the rate of glucose utilization of the cells dropped significantly at 100 μg/ml when compared to the untreated control.

The *in vitro* MTT cytotoxicity assay showed the CAQ and BAQ extracts of *H. petiolare* had no toxic effect on the HepG2 (C3A) cell lines across all concentrations compared to the untreated and metformin-treated controls ( Figures 2b and 2d). The ethanol extract, however, showed significant (p < 0.05) toxicity at 100 μg/ml, where it reduced the cells’ viability by roughly 40% ( Figure 2f).

### α-glucosidase inhibition assay

**Figure 3.**
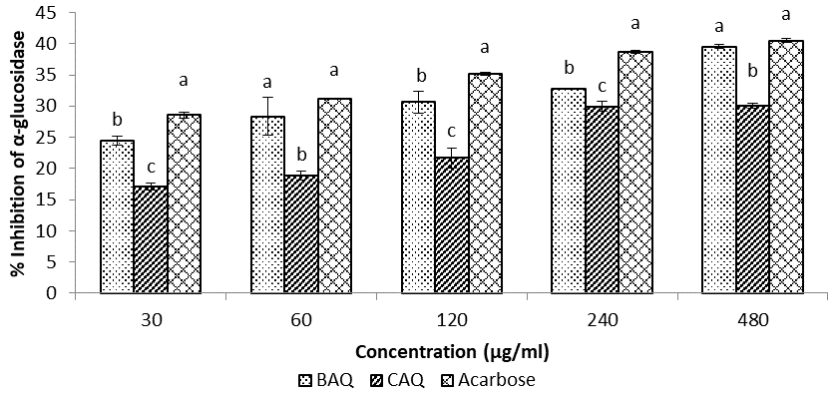
Inhibitory effects of the cold aqueous (CAQ) and boiled aqueous (BAQ) extracts of *H. petiolare* on α-glucosidase enzyme’s activity. Data expressed as mean ± SD (n = 3). Columns with the same alphabet within the same concentration are not significantly different.

Figure 3 shows the inhibition of α-glucosidase activity using the BAQ and CAQ extracts, while Table 1 shows the IC _50_ values of the extracts ^17^. According to the results, both extracts exhibited concentration-dependent inhibition of α-glucosidase activity compared to the standard acarbose (IC _50_ = 804.01 ± 27.09 µg/ml).

**Table 1.** IC _50_ (µg/ml) values of α-amylase, α-glucosidase, and lipase inhibitory activities of the BAQ and CAQ extracts of *H. petiolare.*

Extract	α-glucosidase inhibitory activity IC _50_ (µg/ml)	α-amylase inhibitory activity IC _50_ (µg/ml)	Lipase inhibitory activity IC _50_ (µg/ml)
**BAQ**	844.27 ± 36.81 ^b^	0.361 ± 0.0210 ^a^	117.56 ± 1.66 ^b^
**CAQ**	1075.39 ± 8.46 ^a^	0.383 ± 0.0250 ^a^	228.61 ± 12.73 ^a^
**Acarbose**	804.01 ± 27.09 ^b^	0.378 ± 0.0084 ^a^	-91.00 ± 16.21 ^c^

Values within the same column followed by different superscript are significantly different (p < 0.05).

The inhibitory effects of the BAQ extract (IC _50_ = 844.27 ± 36.81 µg/ml) was, however, significantly (p < 0.05) higher than those of the CAQ extract (IC _50_ = 1075.39 ± 8.46 µg/ml), and significantly closer to those of the standard acarbose across all concentrations.

### α-amylase inhibition assay

**Figure 4.**
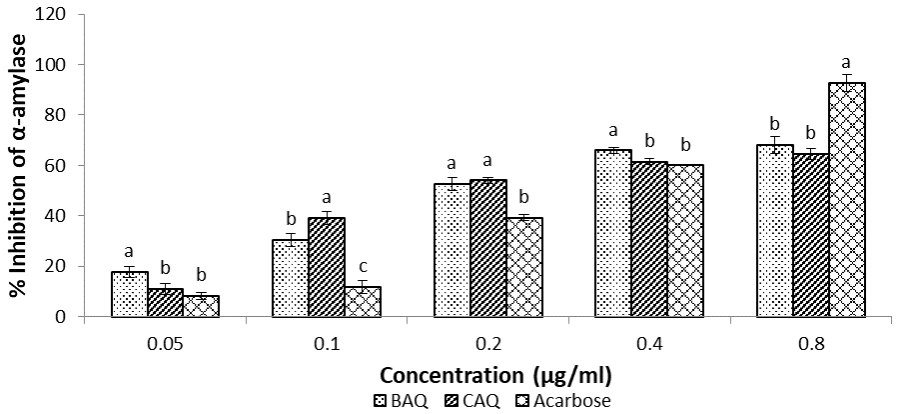
Inhibitory effects of the cold aqueous (CAQ) and boiled aqueous (BAQ) extracts of *H. petiolare* on α-amylase enzyme’s activity. Data expressed as mean ± SD (n = 3). Columns with the same alphabet within the same concentration are not significantly different.

The results of the α-amylase inhibition assay ( Figure 4 and Table 1) showed BAQ and CAQ extracts had concentration-dependent inhibitory effects on α-amylase activity across all concentrations ^17^. The BAQ extract (IC _50_ = 0.361 ± 0.021 µg/ml) had the highest inhibitory effects, even higher than those of the standard acarbose (IC _50_ = 0.378 ± 0.0084 µg/ml) at 0.05 µg/ml and 0.4 µg/ml, respectively. The CAQ (IC _50_ = 0.383 ± 0.025 µg/ml) extract, however, was highest in its inhibitory effect at 0.1 µg/ml and 0.2 µg/ml. The inhibitory effects of both extracts appeared to flatten out from 0.4 µg/ml to 0.8 µg/ml, depicting that there was no apparent increase in the extracts’ inhibitory effects on α-amylase activity at concentrations greater than 0.4 µg/ml.

### Lipase inhibition assay

**Figure 5.**
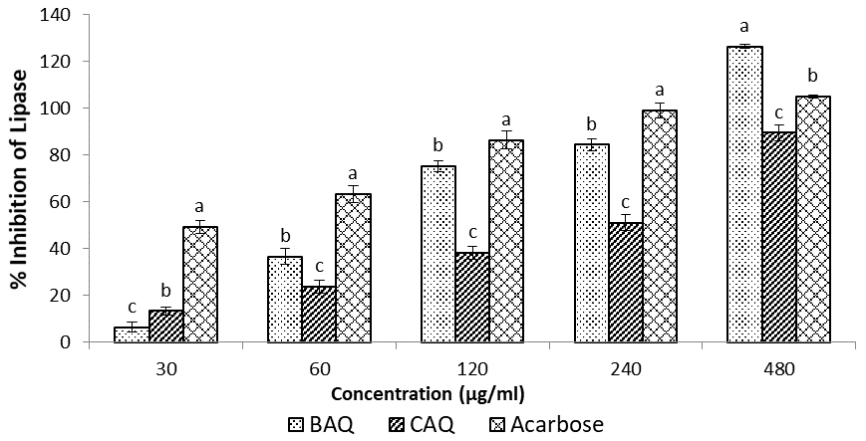
Inhibitory effects of the cold aqueous (CAQ) and boiled aqueous (BAQ) extracts of *H. petiolare* on lipase enzyme’s activity. Data expressed as mean ± SD (n = 3). Columns with the same alphabet within the same concentration are not significantly different.

The results of the lipase inhibition assay using *H. petiolare* BAQ and CAQ extracts, as shown in Figure 5 and Table 1, showed significant concentration-dependent lipase inhibition in both extracts ^17^. The BAQ extract (IC _50_ = 117.56 + 1.66 µg/ml) showed significantly (p < 0.05) higher lipase inhibition when compared with CAQ and the acarbose control; its inhibitory effect rose steadily from being the least at 30 μg/ml (BAQ<CAQ<acarbose), to the highest (BAQ>acarbose>CAQ) at 480 μg/ml. The CAQ (IC _50_ = 228.61 + 12.73 µg/ml) extract, however, displayed the least lipase inhibition across all the concentrations.

## Discussion

Type 2 diabetes mellitus is a metabolic disease that is characterized by insulin resistance and hyperglycaemia. Long-term complications of the disease include macrovascular diseases (e.g. coronary artery disease, and cerebrovascular disease), microvascular diseases (e.g. diabetic retinopathy, and nephropathy), cataracts, erectile dysfunction, infections, diabetic ulcers, etc. ^2^. The disease may be controlled and its complications prevented by bringing down the blood glucose level. This may be achieved through reduction of carbohydrate digestion/absorption and gluconeogenesis, and enhancement of glycogenesis and glucose uptake with minimal side effects and cytotoxicity.

**Figure 6.**
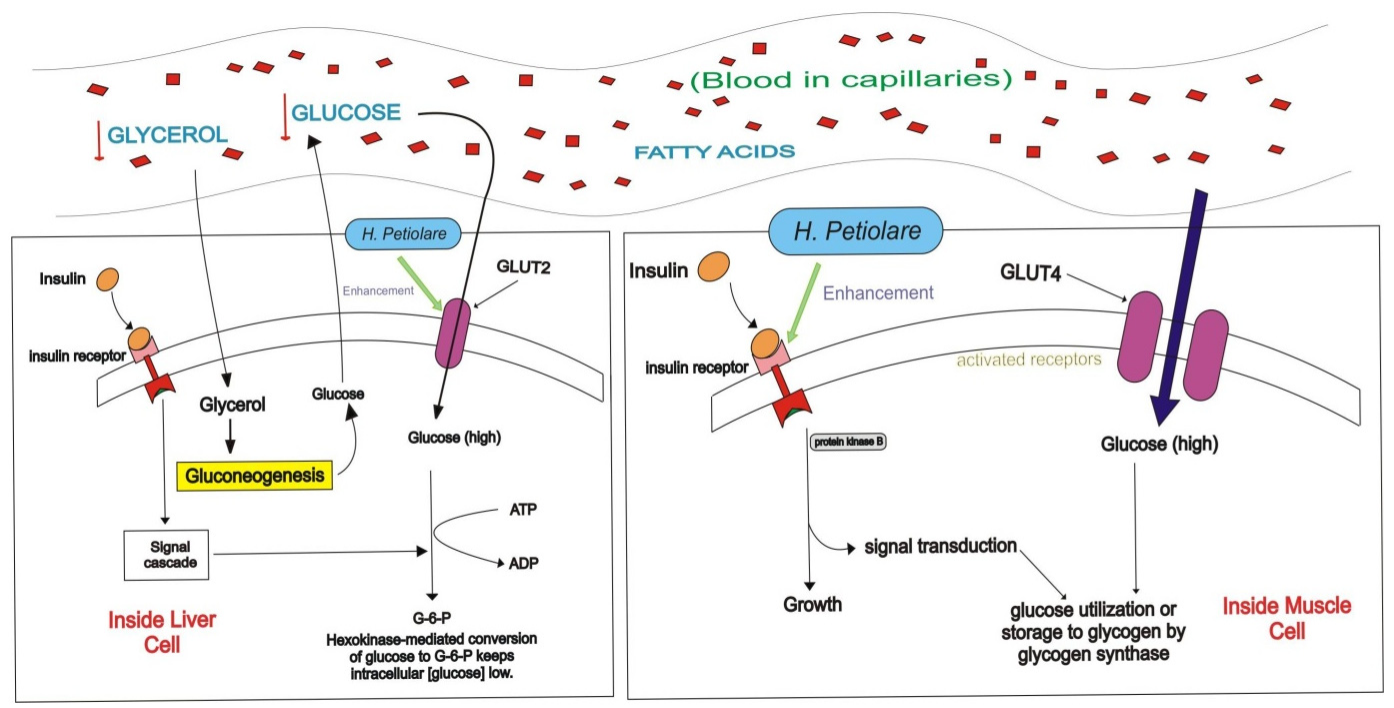
Summary of the proposed effects of *H. petiolare* on glucose uptake in the hepatocytes and myocytes of a human subject. The figure shows the levels of glucose uptake after treatment with *H. petiolare*. “ ↓” indicates decrease.

*H. petiolare*, as shown in this study, could be the effective anti-hyperglycemic agent much needed in achieving this aim. According to our results ( Figure 1f), the ethanol extract of *H. petiolare* showed cytotoxic properties at high concentrations, reducing the viability of the myocytes by more than half at 100 μg/ml. Our previous work showed similar findings, in which the ethanol extract of *H. petiolare* was not only found to be mitotoxic and cytotoxic against C3A hepatocytes, but it was also found to have the potential to induce steatosis and phospholipidosis in the cells ^18^. This further supports the findings of Lourens and co-workers, in their work where the chloroform:methanol (1:1) leaf and stem extracts of *H. petiolare* were found to be toxic against transformed human kidney epithelial (Graham) cells, MCF-7 breast adenocarcinoma, and SF-268 glioblastoma cells ^19^. The cytotoxicity of the ethanol extract reflected its possible myotoxicity in human subjects, and also gave further explanation for the reduced glucose uptake (-50%) observed in the ethanol-treated L6 myocytes at 100 μg/ml ( Figure 1e). The reduced glucose uptake might, however, be the result of a reduced cell population. The CAQ and BAQ extracts showed no cytotoxic effect on the L6 myocytes ( Figure 1b and 1d). The results showed significantly enhanced glucose uptake in the CAQ and BAQ treated L6 cells ( Figure 1a and 1c), most importantly for CAQ. The CAQ extract showed a similar hypoglycaemic effect on the myocytes to the hormone insulin ( Figure 6). Insulin binds to the α-subunit of the tautomeric insulin receptor of the myocyte’s cell membrane, triggering a cascade of reactions which activates the translocation of the GLUT 4 receptor into the membrane to facilitate glucose transport into the cytoplasm ^2^. Phytochemical compounds such as phenols, flavonoids, and flavanols have been reported to facilitate glucose uptake in cells through activation of insulin receptors ^20^. Therefore, the enhanced glucose uptake induced by the extract may be as a result of phytochemicals (i.e. flavonoids, proanthocyanidins, flavonols, saponin, and alkaloids) previously reported by Aladejana *et al*. (2020) to be significantly high in *H. petiolare* ^21^. The CAQ extract may, therefore, be effective in activating insulin receptors, enhancing glucose uptake in the myocytes, and making more glucose available for energy metabolism in exercising muscles; hence, reducing hyperglycaemia and its possible complications, ultimately halting type 2 diabetes.

Cytotoxicity of the ethanol extracts on HepG2 (C3A) hepatocytes as shown in the MTT cytotoxicity assay ( Figure 2f) was also similar to the findings of our previous study ^18^, and suggests the potential hepato- and myotoxicity of the organic extracts of *H. petiolare* in human subjects. The toxicity of the ethanol extract and the consequent reduction in cell viability was possibly responsible for the 61% decrease in glucose utilization observed at 100 μg/ml in the ethanol extract-treated cells ( Figure 2e). The lack of cytotoxicity of the CAQ and BAQ treated HepG2 (C3A) hepatocytes ( Figure 2b and 2d) is reflected in the positive increases in glucose utilization in the cells ( Figure 2a and 2c). However, despite no apparent sign of cytotoxicity, the degree of glucose utilization in the CAQ treated hepatocytes declined by 26% at 100 μg/ml; this may be due to negative feedback as a result of the high concentration of extract. The non-cytotoxicity and significant increases in glucose uptake observed in the BAQ and CAQ treated cells as compared to the metformin-treated control and the untreated control suggests the hypoglycaemic potentials of the extracts, most importantly, the BAQ extract ( Figure 2). The BAQ extract induced hepatic glucose uptake like metformin ( Figure 6). Metformin enhances hepatic glucose uptake by activation of the AMP-activated protein kinase (AMPK), this triggers a series of reactions which may lead to increased liver sensitivity to insulin ^1^. *H. petiolare* is rich in phytochemicals which have been shown by Hanhineva and co-workers to increase hepatic glucose uptake and suppress the hepatic release of glucose ^22^. The BAQ extract may, therefore, be effective in enhancing hepatic glucose uptake in human subjects, triggering glycogenesis or lipogenesis, preventing the hepatic release of glucose, and hence aiding the facilitation of glucose homeostasis.

**Figure 7.**
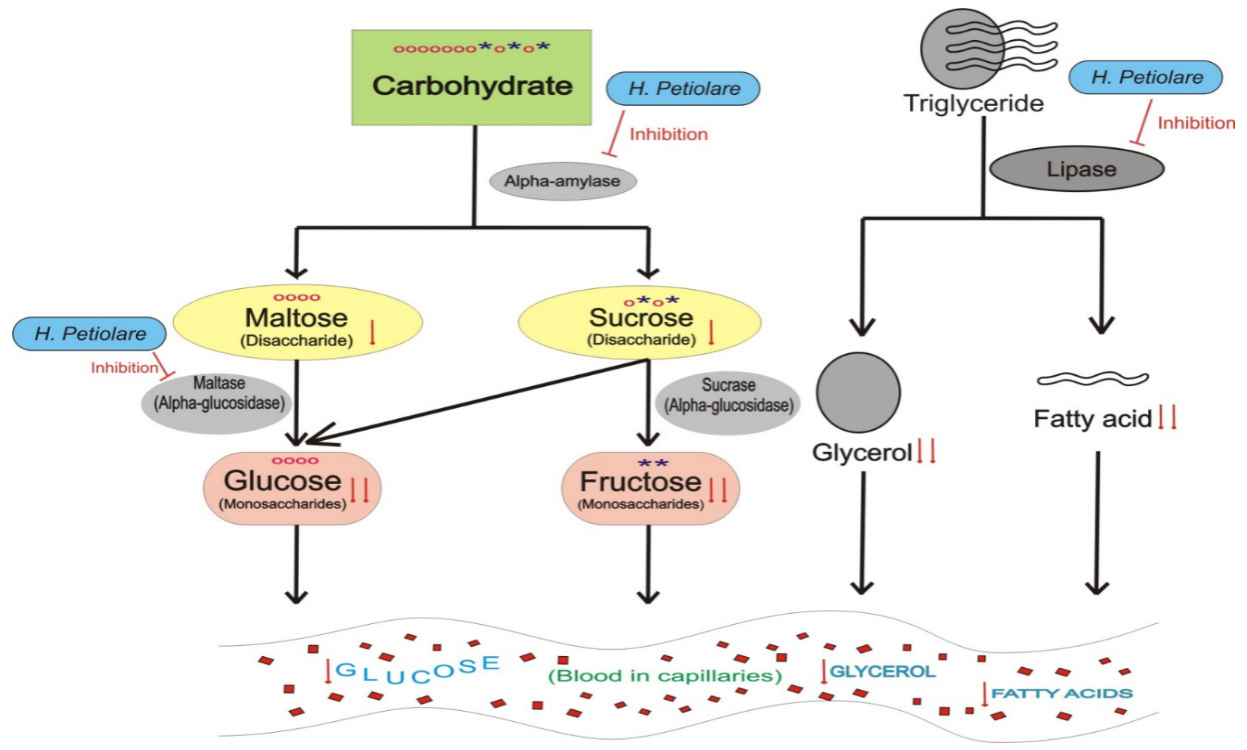
Summarises the possible effects of *H. petiolare* on α-amylase, α-glucosidase, and lipase activities in a human subject. The figure shows the hydrolysis of carbohydrate and triglyceride after treatment with *H. petiolare* respectively. “ ↓” indicates decrease, “ o” represents a glucose molecule, and “*” represents a fructose molecule.

Carbohydrate absorption depends on the presence of amylase, disaccharidases, and normal intestinal mucosal cells with normal active transport mechanisms. The α-amylase enzyme is a glycoside hydrolase that catalyses the hydrolysis of starch at its α-1,4-glycosidic bonds into disaccharides and trisaccharides, which are further converted by other enzymes (e.g. disaccharidases) to glucose ^2^. Alpha-glucosidase (e.g. maltase and sucrose) is a disaccharidase that acts upon α (1→4) bonds. It is found in the brush border of the small intestine where it acts on starch and disaccharides to hydrolyze terminal non-reducing α (1→4)-linked glucose residues to release a single α-glucose molecule ( Figure 7) ^2^. Glucose can be absorbed by the brush-border cells only if all three mechanisms are functioning. Any impairment to one of them may result in reduced or non-absorption of glucose from the intestine, and reduced levels of circulating blood glucose. The ethanol extract of *H. petiolare* was excluded from the enzyme inhibition assay due to its cytotoxicity. Inhibition of α–amylase and α-glucosidase activities by BAQ and CAQ extracts, as shown in the results ( Figure 3 and Figure 4), suggests the extracts (most importantly the BAQ extract) may be effective in modulating postprandial hyperglycaemia by reducing the degree of glucose absorption from the small intestine by enterocytes.

Blood glucose levels are raised in the fasted state through gluconeogenesis, using non-glucose precursors e.g. amino acids, lactate, glycerol etc. Glycerol is derived from the hydrolysis of triglycerides by lipase enzymes. Pancreatic lipase hydrolyses triglycerides at the glycerol/fatty acid bond (primarily in positions 1 and 3) within the duodenum to yield mainly 2-monoglycerides, some diglycerides and free fatty acids ^2^. The yielded glycerol can be taken up by the kidneys or the liver and converted into glucose through gluconeogenesis, thereby elevating the blood glucose level. Inhibition of lipase activity may reduce the rate of gluconeogenesis from the fat precursors and therefore aid in plummeting hyperglycaemia in diabetic patients. The CAQ and BAQ extracts of *H. petiolare* showed concentration-dependent lipase inhibitions ( Figure 5), with the BAQ extract showing significantly higher inhibitory capacity compared to the standard acarbose. This further justifies the use of *H. petiolare* by the traditional healers in the treatment of diabetes and also corroborates the findings of Erasto, Adebola and Afolayan, (2011), and Mahop and Mayet, (2015), who listed *H. petiolare* amongst medicinal plants traditionally used in the treatment of diabetes mellitus ^9, 23^.

## Conclusion

Among all the extracts used, BAQ and CAQ extracts have been shown in this study to have no apparent toxicity to myocytes and hepatocytes. This means either extract can be used in herbal treatment with minimal cytotoxicity on the liver and muscle cells. More studies, however, need to be done to elucidate the effect of the aqueous extracts of *H. petiolare* on the GLUT4 translocation cascade pathway. The whole plant BAQ extract of *H. petiolare* has the biggest effect on glucose uptake in HepG2 (C3A) hepatocytes, and the highest levels of α-amylase, α-glucosidase, and lipase inhibition. The aqueous extracts, more importantly the BAQ extract, may, therefore, contain pharmacologically active hypoglycaemic chemicals, which may be effective in the treatment of diabetes mellitus.

## Data availability

Figshare: *In-vitro* evaluation of the anti-diabetic potential of *Helichrysum petiolare* Hilliard & B.L. Burtt using HepG2 (C3A) and L6 cell lines. https://doi.org/10.6084/m9.figshare.13035002 ^17^.

This project contains the following underlying data:

- A-Amylase.xlsx
- A-Glucosidase Assay.xlsx
- Glucose Utilization C3A.xlsx
- Glucose Utilization L6.xlsx
- Lipase Assay.xlsx

Data are available under the terms of the Creative Commons Zero "No rights reserved" data waiver (CC0 1.0 Public domain dedication).
