# Supplementary material for: In vitro evaluation of the anti-diabetic potential of Helichrysum petiolare Hilliard & B.L. Burtt using HepG2 (C3A) and L6 cell lines
Source: F1000Res. 2021 Apr 1;9:1240. Originally published 2020 Oct 15. [Version 2] doi: 10.12688/f1000research.26855.2 (PMC8080987; doi:10.12688/f1000research.26855.2)
Supplement: Peer review history

# Peer review: Reviewer response for version 2

**DOI:** 10.5256/f1000research.55726.r82718

**Published:** 2021-04-22

**Authors:** Yihong Bao

**Affiliations:** 1 School of Forestry, Northeast Forestry University, Harbin, China

The manuscript has been modified and supplemented in accordance with the amendments, basically meets the requirements, it is recommended to be accepted.

Is the work clearly and accurately presented and does it cite the current literature?

Yes

If applicable, is the statistical analysis and its interpretation appropriate?

Yes

Are all the source data underlying the results available to ensure full reproducibility?

Yes

Is the study design appropriate and is the work technically sound?

Partly

Are the conclusions drawn adequately supported by the results?

Partly

Are sufficient details of methods and analysis provided to allow replication by others?

Partly

Reviewer Expertise:

Development and deep processing of forest resources, enzyme engineering and biotechnology, separation and application of natural active factors, etc.

I confirm that I have read this submission and believe that I have an appropriate level of expertise to confirm that it is of an acceptable scientific standard.

# Peer review: Reviewer response for version 1

**DOI:** 10.5256/f1000research.29657.r79881

**Published:** 2021-03-08

**Authors:** Yihong Bao

**Affiliations:** 1 School of Forestry, Northeast Forestry University, Harbin, China

In this work, the authors explored the anti-diabetic potentials of the ethanol, cold aqueous (CAQ) and boiled aqueous (BAQ) extracts of *Helichrysum petiolare* Hilliard & B.L. Burtt in vitro. They explored the utilization of glucose by L6 myocytes and HepG2 (C3A) hepatocytes under the intervention of different concentrations of extracts. In addition, they also evaluated the inhibitory effect of extracts on α-amylase, α-glucosidase and lipase.

 Specific comments:

1. The Helichrysum petiolare in the keywords should be in italics.
2. When preparing the ethanol extract, what is the concentration of ethanol? 
3. In the section of "Cell culture maintenance", the growth medium is RPMI 1640. There are some contradictions.
4. "The blend was cooled down to optimum temperature and mixed with 5 ml of distilled water". How to control to reach the "optimal temperature"?
5. In the section of ''α-glucosidase inhibition assay'', the article mentioned ''a blank'' and ''sample blank'', why these two groups of data are not reflected in the formula of %inhibition.
6. The author dissolved the extract with DMSO and determined the concentration. After that, the volume was adjusted to 1 mL with Tri-HCl. The concentration of the extract in the system has changed. Should the concentration of the extract be recalculated here?
7. The author consider that concentration-dependent increases in glucose uptake in the BAQ treated cells. However, there are only three concentrations of extracts, and there was no significant difference in the utilization of glucose between the 50 μg/ml and 100 μg/ml. Why the authors didn’t evaluate the inhibitory rate at other concentrations.
8. The abbreviation "ETQ" in the Figure does not appear in the text. It should be explained in the text, not just in the legend.

Is the work clearly and accurately presented and does it cite the current literature?

Yes

If applicable, is the statistical analysis and its interpretation appropriate?

Yes

Are all the source data underlying the results available to ensure full reproducibility?

Yes

Is the study design appropriate and is the work technically sound?

Partly

Are the conclusions drawn adequately supported by the results?

Partly

Are sufficient details of methods and analysis provided to allow replication by others?

Partly

Reviewer Expertise:

Development and deep processing of forest resources, enzyme engineering and biotechnology, separation and application of natural active factors, etc.

I confirm that I have read this submission and believe that I have an appropriate level of expertise to confirm that it is of an acceptable scientific standard, however I have significant reservations, as outlined above.

# Response

**Published:** 2021-03-26

**Authors:** Adebowale Emmanuel Aladejana

**Affiliations:** Medicinal Plant and Economic Development (MPED) Research Centre, University of Fort Hare, Alice, Eastern Cape, South Africa

Dear Yhiong Bao

 Below are responses to your 3rd, 5th, 6th, and 7th comments (response to other comments have been effected in the text):

 3)  We used a method described by Sagbo *et al.*, (2018) and van de Venter *et al.*, (2008), we will appreciate it if you elaborate on any contradiction you observed.

 5)  There was no need to include the sample blank (zero enzyme activity) in the equation since it was used to zero the spectrophotometer.

 6)  No, the enzyme was prepared using Tris-HCl and was then reacted with the extract. This has been corrected in the “pancreatic lipase inhibition” method section.

 7)  The statement “concentration-dependent increases in glucose uptake in the BAQ treated cells” was only made under “Glucose utilization in HepG2 (C3A) hepatocytes” (figure 2c), we believe you are referring to figure 1c under “Glucose utilization assay using L6 myocytes” where we have already stated that BAQ had no concentration-dependent effect on glucose uptake in the cells.

# Peer review: Reviewer response for version 1

**DOI:** 10.5256/f1000research.29657.r79879

**Published:** 2021-03-04

**Authors:** Abimbola Oloye

**Affiliations:** 1 Department of Veterinary Surgery and Theriogenology, College of Veterinary Medicine, Federal University Of Agriculture, Abeokuta, Abeokuta, Nigeria

The study underscores the importance of aqueous extract (Cold and Boiled) of Helichrysum petiolare Hilliard &B.L. Burtt in ameliorating Diabetes type 2 via enhanced glucose uptake and appreciable inhibition of  α–amylase and α-glucosidase. Easy accessibility to the plant by locals and non toxic nature of the aqueous extract make this study pertinent and beneficial. 

 I will like the authors to fill the following gaps:

- In what form is the plant extract consumed by the locals. i.e what medium of extraction do they employ for consumption? If local gin (methanol or ethanol in some cases) is used, then the associated toxicity revealed in this work becomes a vital piece for dissemination to prevent risk to life.
- Can the authors add the Herberium number obtained for the plant to their report?
- The percentage yield of extract from the oven dried plant should be presented. It is expected that the oven dried plant should have been weighed and the yield calculated after the extraction exercise. 
- Finally, I will like to submit that the study was quite revealing. Further studies to unravel and elucidate the pharmacological active factors contained in the plant, which might be responsible for the anti-diabetic properties of the plant, will be highly beneficial.

Is the work clearly and accurately presented and does it cite the current literature?

Yes

If applicable, is the statistical analysis and its interpretation appropriate?

Yes

Are all the source data underlying the results available to ensure full reproducibility?

Yes

Is the study design appropriate and is the work technically sound?

Yes

Are the conclusions drawn adequately supported by the results?

Yes

Are sufficient details of methods and analysis provided to allow replication by others?

Yes

Reviewer Expertise:

Reproductive toxicology

I confirm that I have read this submission and believe that I have an appropriate level of expertise to confirm that it is of an acceptable scientific standard.
